# When Things Go Right: Safety II in an Academic Emergency Department

**DOI:** 10.7759/cureus.70164

**Published:** 2024-09-25

**Authors:** Samantha Boettcher, Jamie Aranda, Ashley Pavlic, Meagan Ladell, Kathleen S Williams, Morgan D Wilbanks, Nancy Jacobson

**Affiliations:** 1 Department of Emergency Medicine, Medical College of Wisconsin, Milwaukee, USA; 2 Department of Pediatrics, Medical College of Wisconsin, Milwaukee, USA

**Keywords:** emergency medicine, graceful extensibility, patient safety, proactive safety, safety ii, work-as-done

## Abstract

Objectives

Historically, “safety” has been defined as an absence of error. Practice variability of any kind has been viewed as a threat. This is termed Safety I. Meanwhile, Safety II posits that clinician practice variability is a necessary adaptation required to meet patient needs in a complex healthcare system. We hypothesize that clinicians in the emergency department (ED) make proactive adaptations and utilize practice variability to enhance safety.

Methods

This is a cross-sectional survey-based study of clinical team members at a tertiary care academic ED with an annual census of 76,000. Qualitative data on instances of proactive safety were collected and analyzed via inductive response analysis. Ratings of support for practice adjustments and experiences of negative emotions during practice variability were collected using 5-point Likert scales.

Results

There were 84 respondents, of which 33 (39%) were nurses, 28 (33%) were physicians, and 23 (27%) held other clinical roles. Qualitative data included 128 instances of harm prevention, with some respondents describing more than one. Instances include proactive safety via collaboration (n = 47; 36.72%), time-based interventions (n = 46; 35.94%), physical harm prevention (n = 27; 21.10%), and patient/visitor interactions (n = 8; 6.25%). Of respondents, 73.17% (n = 30) felt that policies matched daily work most of the time, and 92.68% (n = 38) felt enabled to adapt to the needs of a patient most of the time. Experiences of self-doubt, fear of repercussion, or anxiety occurred most when adhering to a policy despite patient needs (n = 20; 48.78%).

Conclusions

Clinical team members report keeping patients safe through various forms of behavior adaptations, most frequently reporting collaboration beyond usual expected practice. Respondents report policies to match daily work most of the time and feel enabled to adapt to the needs of patients. Feelings of self-doubt, fear of repercussion, or anxiety were reported most frequently when clinicians adhered to a policy despite the needs of a patient.

## Introduction

When the Institute of Medicine released To Err is Human: Building a Safer Health System in 1999, it brought new attention to the burden of medical error and was a call to action to address patient safety [1]. Since that time, there have been varying approaches to safety borrowed from industry and from human factors scientists [2]. The Agency for Healthcare Research and Quality defines patient safety as “the prevention of errors, injury, or other preventable harm and reduction of unnecessary harm [3]”, and the WHO states that patient safety “aims to prevent and reduce risks, errors, and harm that occur to patients [4].” This philosophy on errors has generated a “find and fix” approach of retrospective detection and investigation of past events with a focus on reducing the burden of errors. In this way, “safety” is defined by what it is not and is studied only in its absence. In From Safety-I to Safety-II: a white paper, Hollnagel et al. term this retrospective and reactive approach to safety as “Safety I” [2]. Practical implementation of this approach results in drawing reductionist linear cause-and-effect relationships between error and outcome, assuming that deviation from standard practice introduces risk, and failing to learn from (or ignoring) the vast majority of cases in which adaptive actions by clinicians actually keep patients safe [5]. By examining only instances of undesired outcomes and noting clinician practice variability, we have historically failed to recognize that the same practice variability is present during daily expected work and also results in exceptional outcomes. Indeed, adaptive practice variability is not only ubiquitous but also necessary to deliver patient care in our complex healthcare system.

Hollnagel offers a novel approach to patient safety, termed Safety II. This approach shifts the paradigm away from avoidance of errors and toward the promotion of systems functioning to achieve intended outcomes under varying conditions [2]. Safety II posits that performance variability is necessary and clinical experts making dynamic practice adjustments within a complex system serve as a vital source of flexibility. Safety II, in contrast to Safety I, is prospective and anticipatory. It aims to understand how systems and people achieve desired outcomes most of the time [2]. Healthcare delivery has an infinite number of variables occurring with team, patient, and sociotechnical system factors. In most cases, desirable outcomes are achieved despite complex systems and nuanced situations. The concept of “graceful extensibility” is defined as a system’s or individual’s ability to adapt to unexpected stressors or disruptions, and it is essential for patient safety [2,6,7]. Although Safety I suggests that variability in healthcare increases the risk of harm, Safety II embraces the concept of graceful extensibility and proposes that individuals working within a complex system must make real-time adjustments in order to meet the needs of nuanced situations and provide effective care [2,6-8]. The differences in approach between Safety I and Safety II generate tension between proposed solutions.

Historic efforts to improve safety have often included implementing policies to restrict behaviors to achieve a desirable outcome. However, attempts to minimize undesired outcomes have instead introduced barriers to achieving desired outcomes. These barriers emerge from failure to account for the innumerable situation-specific variables that may arise. When examining this phenomenon, one must consider the difference between work-as-imagined and work-as-done [2,6-7]. Work-as-imagined is the perception of working within a system that is understood by those designing the system. Work-as-done is the lived experiences of those tasked with achieving desired outcomes within that system [2]. Well-intended policies implemented in the context of a gap between work-as-imaged and work-as-done often generate further barriers to safe and efficient achievement of desired outcomes [2,6,8]. Rather than promoting desired behaviors, these policies often result in decreased efficacy, as individuals employ workarounds to meet the needs of situations not included in work-as-imaged. In this way, proposed solutions often inhibit graceful extensibility, thus negatively impacting most cases where expert adaptations keep patients safe. Therefore, historically employed solutions may actually increase the risk of harm [2,6-8]. In contrast to Safety I solutions that attempt to approximate work-as-done to work-as-imagined, Safety II solutions instead attempt to better understand and enable work-as-done, including necessary practice adaptations [2].

The reality of work-as-done in the emergency department (ED) requires constant adjustments to novel and unpredictable situations. Therefore, allowance of performance variability and graceful extensibility are of particular importance in this setting. In this survey-based study, we aimed to explore ways in which ED care team members employ practice variability to meet the needs of unique clinical situations and if their experience of adaptive behaviors prompted anxiety, self-doubt, or fear of repercussion. We additionally aimed to evaluate if ED care team members perceive a gap between work-as-imaged and work-as-done in our clinical practice environment.

## Materials and methods

Study design and population

This is a cross-sectional survey-based study of all healthcare team members in patient-facing roles at our 55-bed, tertiary care, urban academic ED, and level one trauma center with an average annual census of 76,000. This study and methodology were approved by our institutional internal review board (PRO00040367).

Survey content and administration

The survey was created de novo by institutional leaders, including physicians with advanced knowledge in quality and safety and human factors engineers familiar with Safety I and Safety II (Appendix A). Responses were anonymous, and no respondent or patient-identifying information was collected. All patient-facing team members were invited to respond, including emergency medicine (EM) attendings, EM fellows, EM residents, rotating medical students, physician assistants and nurse practitioners, pharmacists, respiratory therapists, nurses, technicians, health unit coordinators, and social workers. This anonymous survey was sent in July 2022 from a human factors engineer, who was considered an impartial third party with minimal to no influence on survey participants. All team members were sent an invitation to participate via email with an informational letter detailing the risks, benefits, and voluntary nature of participating in this study. Given the sensitive topic of the survey content, we additionally provided information on resources if feelings of self-doubt, fear of repercussion, or anxiety arose from answering questions in the survey. The invitations to participate, the survey, and an informational letter were re-sent via email every 30 days for two additional cycles. Responses were collected for 90 days following the last invitation to respond.

Survey content

The survey had 10 questions and took about 10 minutes to complete. Demographic data was collected, including healthcare team roles, years of clinical experience, age, and self-reported gender. Respondents were asked to describe, utilizing free text, an instance when they had acted or witnessed another team member acting to keep a patient safe. Respondents were asked to describe a time when they or a team member bypassed a current policy or protocol to meet the needs of a patient or situation. Respondents utilized a 5-point Likert scale to rate the following prompts: “My work environment allows enough independence for me to adapt to the needs of patients or situations,” “I have to bypass current policies or protocols to meet the needs of a patient or situation,” and “Policies and protocols reasonably match my day-to-day work.” Respondents utilized a 5-point Likert scale to report feelings of “self-doubt, fear of repercussion, or anxiety” when (1) bypassing a current policy or protocol in order to meet the needs of a patient or situation; (2) strictly adhering to a current policy or protocol despite the needs of a patient or situation; and (3) encountering a lack of current policy or protocol.

Data analysis

Demographic data was analyzed via descriptive statistics. Qualitative data was analyzed via inductive response analysis based on grounded theory. Submissions were categorized into themes and then reconciled into overarching classifications until theoretical saturation was achieved. Coding was conducted, and determinations were made based on author consensus. Sometimes, respondents provided more than one example, and these were coded independently. However, each example was only coded into one theme. Exemplative responses, themes, and categories can be found in the Results section. Likert scale data was analyzed descriptively. Additionally, the Mann-Whitney U test was utilized for comparative analysis based on role and gender, and the Kruskal-Wallis test was used for comparative analysis based on age and years in clinical practice. A p-value of <0.05 was considered statistically significant.

## Results

Demographic data

There were 84 total survey respondents. Of the respondents, 39.29% (n = 33) were nurses, 33.33% (n = 28) were physicians (attendings, fellows, and residents), and the remaining 27.38% (n = 23) included other patient-facing roles such as technician/technologists, medical students, and physician assistants and nurse practitioners. The age range distributions of respondents were as follows: 26.19% (n = 22) were 20-29 years old, 52.28% (n = 44) were 30-39 years old, 17.86% (n = 15) were 40-49 years old, and 3.57% (n = 3) were 50-59 years old. When analyzing years of experience in clinical settings, 28.57% (n = 24) had 0-5 years, 41.67% (n = 35) had 6-10 years, 11.90% (n = 10) had 11-15, 10.71% (n = 9) had 16-20 years, and 7.14% (n = 6) had more than 20 years. Females comprised 63.10% (n = 53) of respondents, males made up 34.52% (n = 29), and those who identified as non-binary were 2.38% (n = 2) of respondents. These results are summarized in Table 1. The 84 respondents provided 127 descriptions of keeping a patient safe and 42 descriptions of bypassing a policy or expected workflow to meet the needs of a patient or situation.

**Table 1:** Descriptive statistics of respondent characteristics The data has been represented as the number of responses and percent of total responses (n, %).

Variable	n (%)
Care team role	
Nurse	33 (39.29%)
Attending physician	19 (22.62%)
Resident or fellow physician	9 (10.71%)
Technician/technologist	8 (9.52%)
Medical student	7 (8.33%)
Advanced practice provider	3 (3.57%)
Other clinical role	2 (2.38%)
Other non-clinical patient-facing roles	2 (2.38%)
Age	
20-29	22 (26.19%)
30-39	44 (52.38%)
40-49	15 (17.86%)
50-59	3 (3.57%)
Years in clinical practice	
0-5	24 (28.57%)
6-10	35 (41.67%)
11-15	10 (11.90%)
16-20	9 (10.71%)
Over 20	6 (7.14%)
Gender	
Female	53 (63.10%)
Male	29 (34.52%)
Non-binary	2 (2.38%)

Qualitative data set 1: adjusting behavior to keep a patient safe

**Figure 1:**
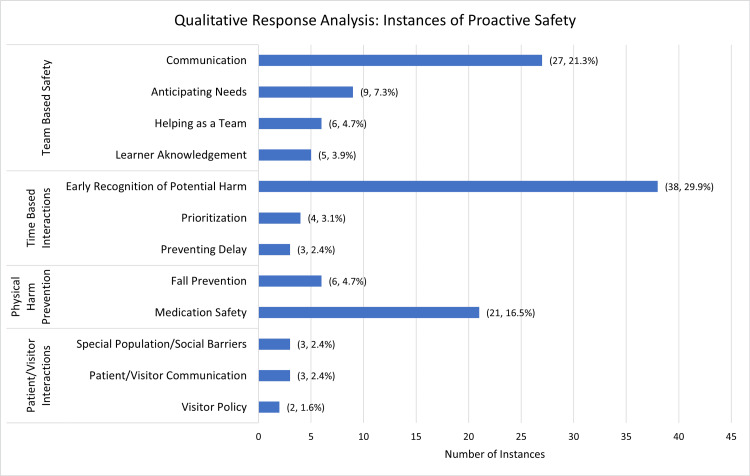
Responses detailing instances of proactive safety were grouped into themes and consolidated into overarching categories through inductive response analysis Respondents most frequently described time-based interventions. The data is reported as an absolute number of reported instances and percent of total reported instances (n, %).

The 84 survey respondents provided 127 descriptions of care team members making real-time adjustments to ensure patient safety. These instances were grouped into 12 themes: early recognition of potential harm (n = 38; 29.92%), triaging and prioritizing of tasks (n = 4; 3.15%), preventing delay (n = 3; 2.36%), team communication (n = 27; 21.26%), anticipating the needs of another team member (n = 9; 7.09%), helping a team member keep a patient safe (n = 6; 4.72%), recognizing that a care team members as learners (n = 5; 3.94%), medication safety (n = 21; 16.54%), fall prevention (n = 6; 4.72%), special populations/social barriers (n = 3; 2.36%), patient and visitor communication (n = 3; 2.36%), and visitor policies (n = 2; 1.57%). The themes were then reconciled into overarching categories, which included team-based collaboration (n = 47; 36.72%), time-based interventions (n = 46; 35.94%), physical harm prevention (n = 27; 21.10%), and patient or visitor interactions (n = 8; 6.25%) (Table 2, Figure 1).

**Table 2:** Qualitative responses illustrating instances where clinical team members ensured patient safety Responses were analyzed via inductive response analysis. They were grouped into themes and then reconciled into overarching categories. A total of 127 described instances were analyzed (n = 127). The data has been represented as the number of responses and percent of total responses (n, %).

Category	Theme	Exemplative response
Team-based collaboration (n = 47; 37.01%)	Team communication (n = 27; 21.26%)	I clearly communicated and then confirmed our plan of care for the patient. I ensured nursing staff knew what we were doing and why. We confirmed doses and routes of medications — what I thought the underlying problem was.
	Anticipating the needs of a team member (n = 9; 7.09%)	A patient arrives with a clinical presentation of a dissection. Providers were busy with a cardioversion next door. Writer and nursing team double-lined, labs drawn, EKG printed US bedside. The physician was pulled from next door due to critical status. The FAST exam was positive. The patient went to the resuscitation bay.
	Helping a team member keep patients safe (n = 6; 4.72%)	I was assigned to the trauma room but did not have a patient assigned to me, so I was rounding through the teams while the arena nurses were 4:1 due to low staffing.
	Recognizing a team member as a learner (student or resident) (n = 5; 3.94%)	There are times that orders are put in by residents, and I will double check if it seems like it is not correct with the attending.
Time-based interventions (n = 45; 35.43%)	Early recognition of potential harm (n = 38; 29.92%)	I was called to a patient’s bedside for acute respiratory distress. CXR demonstrated a pneumothorax. I loudly stated I was going to perform an L-sided needle decompression but was prepping the patient’s R anterior chest. A PA observing at the back of the room stopped the procedure.
	Triaging and prioritizing of tasks (n = 4; 3.15%)	The patient came in by EMS, and the doctor was outside helping another patient. I went out and urgently informed the doctor that a new sick patient had just arrived.
	Preventing delay (n = 3; 2.36%)	Calling every ambulance company to see who has the quickest ETA.
Physical harm prevention (n = 27; 21.26%)	Medication safety (n = 21; 16.54%)	I double-checked my lidocaine (for digital block) and found the nurse had pulled a bottle of etomidate.
	Fall prevention (n = 6; 4.72%)	We saw a patient almost roll off the bed, but we put the rails up before they could fall off.
Patient/visitor interaction (n = 8; 6.30%)	Recognition of barriers and special populations (n = 3; 2.36%)	The patient was an IV drug user and had previously been sex trafficked. The patient was activated, and her POA was her boyfriend. However, the team was concerned that this boyfriend was involved in her sex trafficking. Therefore, as a team RN, social worker, resident, and attending, we made the decision to place the patient confidential.
	Patient and visitor communication (n = 3; 2.36%)	There was a well-known patient who tended to be violent. I asked unnecessary bystanders to evacuate the area and asked security if it would be okay if they could just be out of sight of the patient but nearby in case, they escalated. I noticed that the patient tends to be uncooperative when they are overstimulated and have many people speaking to them all at once. The patient agreed to medical interventions and remained calm the entire stay.
	Visitor policies (n = 2; 1.57%)	Nurses have bypassed the current visitor policy: (1) removed visitors from the room to keep the patient calm and safe and (2) brought in extra visitor(s) for patient comfort.

Qualitative data set 2: bypassing a policy or workflow

**Figure 2:**
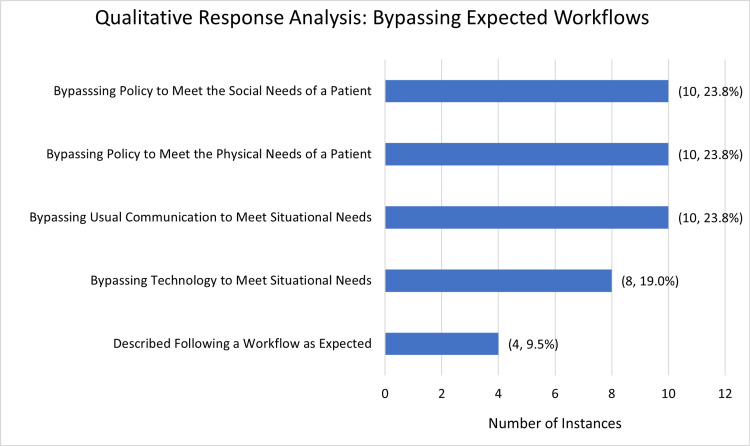
Responses detailing instances where clinical team members bypassed a policy or standard workflow to meet patient or situational needs were grouped into four themes through inductive response analysis The data is reported as an absolute number of reported instances and percent of total reported instances (n, %).

Respondents also described 42 examples of bypassing a policy or workflow to keep a patient safe. These instances were coded into four different themes: bypassing policies to meet the physical needs of a patient (n = 10; 23.81%), bypassing policies to meet the behavioral or social needs of a patient (n = 10; 23.81%), communication outside of normal routes to meet the needs of a situation (n = 10; 23.81%), and bypassing usual technology-based workflow (n = 8; 19.05%). In 9.52% (n = 4) of instances, the respondent described a situation in which a protocol was followed. Results and example responses can be found in Table 3. Given the relatively smaller number of descriptions and fewer themes, these responses were not reconciled into larger categories (Figure 2).

**Table 3:** Qualitative responses highlighting instances where clinical team members bypassed a policy or standard workflow to address patient needs or adapt to specific situations Responses were analyzed via inductive response analysis. A total of 42 described instances were analyzed (n = 42). The data has been represented as the number of responses and percent of total responses (n, %).

Theme	Exemplative response
Meeting the physical needs of a patient (n = 10; 23.81%)	A patient wouldn’t stay for a stroke workup unless they could smoke. I let them go outside and smoke.
Meeting the behavioral or social needs of a patient (n = 10; 23.81%)	A behavioral health patient had come in with concerning ideations. Per protocol, visitors are not allowed with the patient during assessment. A provider allowed a family member to be beside them, as that was the patient’s support system and was an asset to the care team.
Meeting the needs of a situation by communication outside of normal routes (n = 10; 23.81%)	The patient was here due to pain from an unremoved ex-fix. The ortho and hospitalist both didn’t want to admit the patient for pain control, and the ortho refused to even see the patient — I ended up calling (the ED medical director) for rescue.
Meeting the needs of a situation by bypassing usual technology-based workflow (n = 8; 9.52%)	I had a hypoglycemic patient who required D50 during a power outage. The Pyxis machine for dispensing medications on my team was not working (possibly plugged into the wrong outlet?). A nurse had to go to another patient care area and override the medication to get dextrose to prevent further patient decompensation.
Described an instance of following an expected workflow (n = 4, 9.25%)	In the ED, we had to sedate a patient to prevent them from physically harming themselves.

Likert scale data: adaptive variability and work-as-imagined versus work-as-done

**Figure 3:**
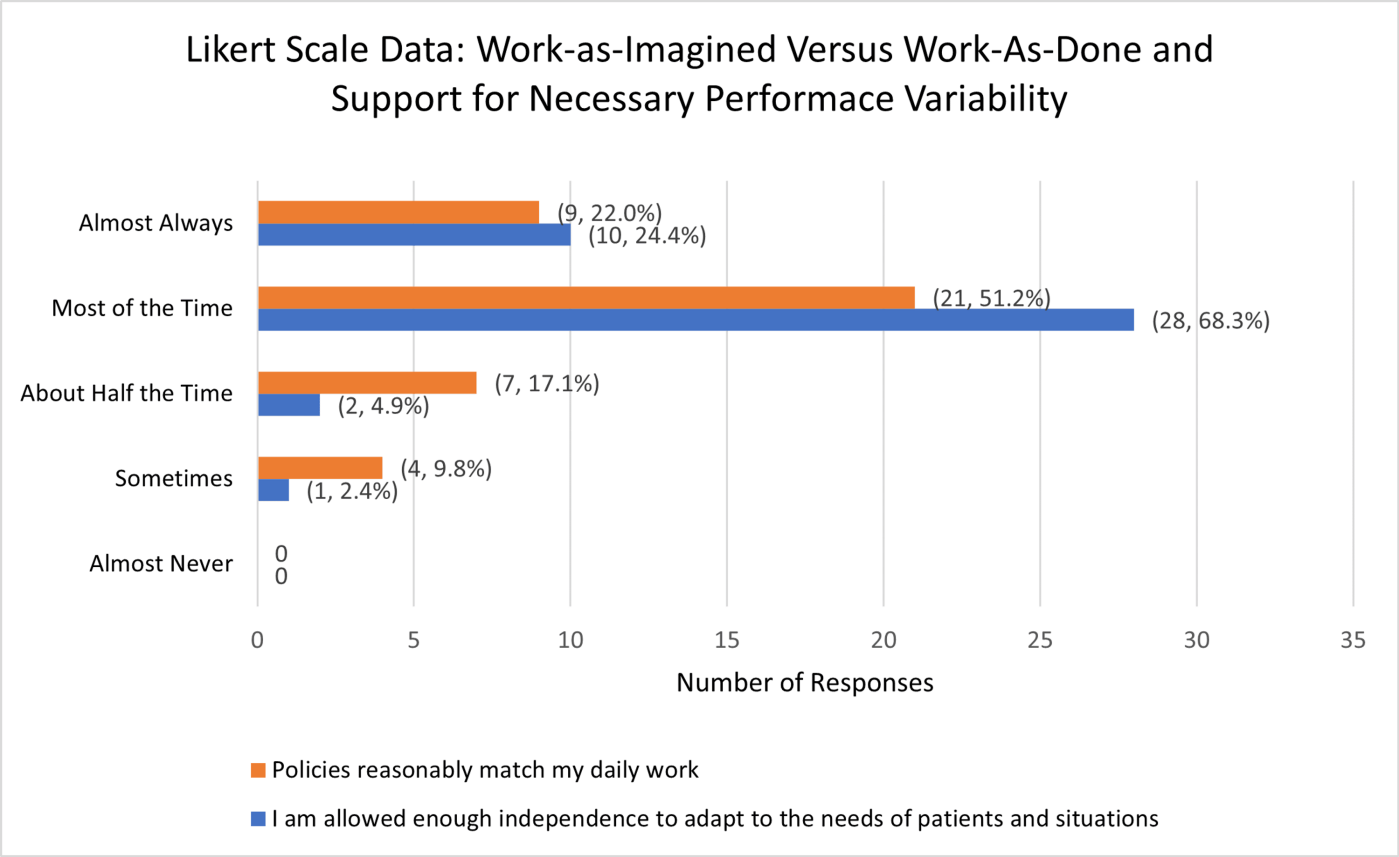
Likert scale data: work-as-imagined versus work-as-done and support for necessary performance variability Respondents rated utilizing 5-point Likert scales to report perceptions of policies matching daily work and support for practice variability. Most respondents felt policies matched their daily work most of the time or almost always and felt supported most of the time or almost always. A total of 41 responses were recorded (n = 41). The data has been represented as the number of responses and percent of total recorded responses (n, %).

Of 84 total respondents, 41 completed the Likert scale data. When asked whether their work environment allowed their independence to adapt, 73.17% (n = 30) responded that policies and protocols matched day-to-day work “most of the time” or “almost always.” When respondents were asked if felt they were able to adapt to the needs of a patient or situation, 92.68% (n = 38) responded “most of the time” or “almost always.” No respondents reported “almost never” for either prompt (Figure 3).

Likert scale data: emotional responses to exercising practice variability

**Figure 4:**
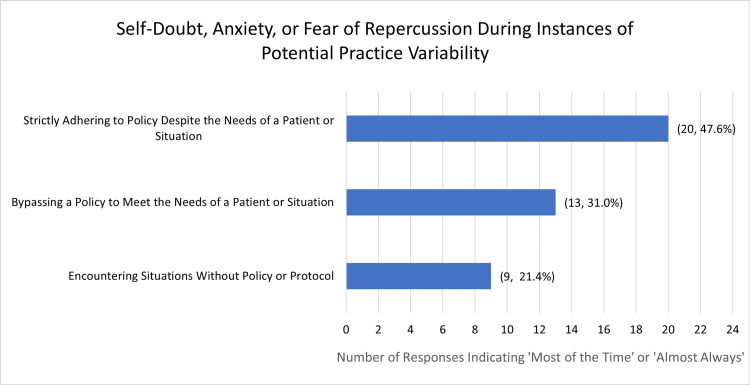
Experiences of self-doubt, anxiety, or fear of repercussion during instances of potential practice variability Respondents utilized a 5-point Likert scale to report how often they experience self-doubt, anxiety, or fear of repercussion. Respondents most frequently reported feeling anxiety “most of the time” or “almost always” when strictly adhering to policies despite the needs of their patients. A total of 42 responses were recorded (n = 42). The data has been represented as the number of responses and percent of total responses (n, %).

Of all respondents, 42 submitted responses to this data set. Experiences of negative emotion (self-doubt, fear of repercussion, or anxiety) were present when strictly adhering to policies, bypassing policies, or encountering situations where no policy existed. Specifically, 48.78% (n = 20) of respondents reported these feelings “almost always” or “always” when strictly adhering to a policy despite the needs of a patient, 41.47% (n = 17) when encountering a situation without a policy, and 32.50% (n = 13) when bypassing a policy to meet the needs of a patient. Some respondents reported experiencing these emotions in more than one of the scenarios and 42.26% (n = 17) of respondents reported never experiencing self-doubt, fear of repercussion, or anxiety for any of the stated scenarios (Figure 4).

Comparative data

Comparative statistics were performed on Likert scale questions. A p-value of <0.05 was considered statistically significant.

When comparing physician and non-physician responses, there was not a statistically significant difference between responses to the prompts “My work environment allows enough independence for me to adapt to the needs of patients or situations” (W = 223, p = 0.55) or “Policies and protocols reasonably match day-to-day work” (W = 181, p = 0.52). Likewise, there were no statistically significant differences between physician and non-physician responses regarding experiences of self-doubt, fear or repercussion, or anxiety when bypassing a policy to meet the needs of a patient (W = 198, p = 0.96), when strictly adhering to a policy despite the needs of a patient (W = 206, p = 0.97), or when encountering a lack of policy (W = 214, p = 0.53) (Table 4).

**Table 4:** Comparative analysis of physician vs. non-physician responses The Mann-Whitney U test was used for analysis, and no significant difference was detected for any of the survey prompts. Data is reported as the number of responses and percent of total responses (n (%)). Comparative statistics are reported as W-values and p-values. A p-value <0.05 was considered significant.

Comparative analysis of physician vs. non-physician responses			
“My work environment allows enough independence for me to adapt to the needs of patients or situations.”			
Response	Physician, n (%)	Non-physician, n (%)	Comparative analysis
Almost always	3 (17.65)	7 (29.17)	W = 223, p = 0.55
Most of the time	13 (76.47)	15 (62.50)	
About half the time	0 (0.00)	2 (8.33)	
Sometimes	1 (5.88)	0 (0.00)	
Almost never	0 (0.00)	0 (0.00)	
Total	17 (100)	24 (100)	
Median (IQR)	4 (0)	4 (1)	
“Policies and protocols reasonably match day-to-day work.”			
Response	Physician, n (%)	Non-physician, n (%)	Comparative analysis
Almost always	3 (17.65)	6 (25.00)	W = 181, p = 0.52
Most of the time	12 (70.59)	9 (37.50)	
About half the time	0 (0.00)	7 (29.17)	
Sometimes	2 (11.76)	2 (8.33)	
Almost never	0 (0.00)	0 (0.00)	
Total	17 (100)	24 (100)	
Median (IQR)	4 (0)	4 (1.25)	
“I experience self-doubt, fear of repercussion, and/or anxiety when I bypass a current policy or protocol in order to meet the needs of a patient or situation.”			
Response	Physician, n (%)	Non-physician, n (%)	Comparative analysis
Almost always	1 (5.88)	5 (21.74)	W = 198, p = 0.96
Most of the time	5 (29.41)	2 (8.70)	
About half the time	4 (23.53)	6 (26.09)	
Sometimes	5 (29.41)	7 (30.43)	
Almost never	2 (11.76)	3 (13.04)	
Total	17 (100)	23 (100)	
Median (IQR)	3 (2)	3 (2)	
“I experience self-doubt, fear of repercussion, and/or anxiety when I strictly adhere to a current policy or protocol despite the needs of a patient or situation.”			
Response	Physician, n (%)	Non-physician, n (%)	Comparative analysis
Almost always	3 (12.50)	1 (5.88)	W = 206, p = 0.97
Most of the time	8 (33.33)	8 (47.06)	
About half the time	3 (12.50)	1 (5.88)	
Sometimes	8 (33.33)	5 (29.41)	
Almost never	2 (8.33)	2 (11.76)	
Total	24 (100)	17 (100)	
Median (IQR)	4 (2)	3 (2)	
“I experience self-doubt, fear of repercussion, and/or anxiety when I encounter situations not addressed by current policies or protocols.”			
Response	Physician, n (%)	Non-physician, n (%)	Comparative analysis
Almost always	0 (0.00)	1 (4.17)	W = 214, p = 0.53
Most of the time	2 (12.50)	6 (25.00)	
About half the time	3 (18.75)	3 (12.50)	
Sometimes	8 (50.00)	9 (37.50)	
Almost never	3 (18.75)	5 (20.83)	
Total	16 (100)	24 (100)	
Median (IQR)	2 (1)	2 (2)	

When analyzing responses among age groups, there was not a statistically significant difference in responses to the prompts “My work environment allows enough independence for me to adapt to the needs of patients or situations” (X^2^ = 0.03, df = 2, p = 0.98) or “Policies and protocols reasonably match day-to-day work” (X^2^ = 2.08, df = 2, p = 0.35). There were no statistically significant differences among age groups regarding feelings of self-doubt, fear or repercussion, or anxiety when bypassing a policy or protocol in order to meet the needs of a patient (X^2^ = 0.13, df = 2, p = 0.94) when strictly adhering to a policy or protocol despite the needs of a patient (X^2^ = 0.39, df = 2, p = 0.82), or when encountering a lack of policy or protocol (X^2^ = 0.09, df = 2, p = 0.96). The 40-49 years and over 50 age groups were combined for analysis due to small individual group sizes (Table 5).

**Table 5:** Comparative analysis among age group The Kruskal-Wallis test was used for analysis, and no significant difference was detected for any of the survey prompts. Data is reported as the number of responses and percent of total responses (n (%)). Comparative statistics are reported as X2, df, and p-values. A p-value <0.05 was considered significant.

Comparative analysis among age groups					
“My work environment allows enough independence for me to adapt to the needs of patients or situations.”					
Response	20-29 years, n (%)	30-39 years, n (%)	40-49 years, n (%)	>50 years, n (%)	Comparative statistics
Almost always	2 (16.67)	6 (27.27)	1 (20.00)	1 (50.00)	X^2^ = 0.03, df = 2, p = 0.98
Most of the time	10 (83.33)	14 (63.64)	4 (80.00)	0 (0.00)	
About half the time	0 (0.00)	1 (4.55)	0 (0.00)	1 (50.00)	
Sometimes	0 (0.00)	1 (4.55)	0 (0.00)	0 (0.00)	
Almost never	0 (0.00)	0 (0.00)	0 (0.00)	0 (0.00)	
Total	12 (100)	22 (100)	5 (100)	2 (100)	
Median (IQR)	4 (0)	4 (0.75)	4 (0.50)		
“Policies and protocols reasonably match day-to-day work.”					
Response	20-29 years, n (%)	30-39 years, n (%)	40-49 years, n (%)	>50 years, n (%)	Comparative statistics
Almost always	5 (41.67)	2 (9.09)	2 (40.00)	0 (0.00)	X^2^ = 2.08, df = 2, p = 0.35
Most of the time	4 (33.33)	14 (63.64)	2 (40.00)	1 (50.00)	
About half the time	3 (25.00)	4 (18.18)	0 (0.00)	0 (0.00)	
Sometimes	0 (0.00)	2 (9.09)	1 (20.00)	1 (50.00)	
Almost never	0 (0.00)	0 (0.00)	0 (0.00)	0 (0.00)	
Total	12 (100)	22 (100)	5 (100)	2 (100)	
Median (IQR)	4 (1.25)	4 (0.75)	4 (1.50)		
“I experience self-doubt, fear of repercussion, and/or anxiety when I bypass a current policy or protocol in order to meet the needs of a patient or situation.”					
Response	20-29 years, n (%)	30-39 years, n (%)	40-49 years, n (%)	>50 years, n (%)	Comparative statistics
Almost always	2 (16.67)	2 (9.52)	1 (20.00)	1 (50.00)	X^2^ = 0.13, df = 2, p = 0.94
Most of the time	1 (8.33)	5 (23.81)	1 (20.00)	0 (0.00)	
About half the time	3 (25.00)	6 (28.57)	1 (20.00)	0 (0.00)	
Sometimes	5 (41.67)	6 (28.57)	1 (20.00)	0 (0.00)	
Almost never	1 (8.33)	2 (9.52)	1 (20.00)	1 (50.00)	
Total	12 (100)	21 (100)	5 (100)	2 (100)	
Median (IQR)	2.5 (1.25)	3 (2)	3 (3)		
“I experience self-doubt, fear of repercussion, and/or anxiety when I strictly adhere to a current policy or protocol despite the needs of a patient or situation.”					
Response	20-29 years, n (%)	30-39 years, n (%)	40-49 years, n (%)	>50 years, n (%)	Comparative statistics
Response	20-29 (%)	30-39 (%)	40-49 (%)	50-59 (%)	X^2^ = 0.39, df = 2, p = 0.82
Almost always	1 (8.33)	3 (13.64)	0 (0.00)	0 (0.00)	
Most of the time	4 (33.33)	8 (36.36)	3 (60.00)	1 (50.00)	
About half the time	1 (8.33)	2 (9.09)	1 (20.00)	0 (0.00)	
Sometimes	5 (41.67)	8 (36.36)	0 (0.00)	0 (0.00)	
Almost never	1 (8.33)	1 (4.55)	1 (20.00)	1 (50.00)	
Total	12 (100)	22 (100)	5 (100)	2 (100)	
Median (IQR)	2.5 (2)	3.5 (2)	4 (2)		
“I experience self-doubt, fear of repercussion, and/or anxiety when I encounter situations not addressed by current policies or protocols.”					
Response	20-29 years, n (%)	30-39 years, n (%)	40-49 years, n (%)	>50 years, n (%)	Comparative statistics
Response	20-29 (%)	30-39 (%)	40-49 (%)	50-59 (%)	X^2^ = 0.09, df = 2, p = 0.96
Almost always	1 (9.09)	0 (0.00)	0 (0.00)	0 (0.00)	
Most of the time	3 (27.27)	4 (18.18)	0 (0.00)	1 (50.00)	
About half the time	0 (0.00)	4 (18.18)	1 (20.00)	1 (50.00)	
Sometimes	4 (36.36)	11 (50.00)	2 (40.00)	0 (0.00)	
Almost never	3 (27.28)	3 (13.64)	2 (40.00)	0 (0.00)	
Total	11 (100)	22 (100)	5 (100)	2 (100)	
Median (IQR)	2 (2.5)	2 (1.0)	2 (1.5)		

When analyzing responses between years in clinical practice, there was not a statistically significant difference in response to the prompt “My work environment allows enough independence for me to adapt to the needs of patients or situations” (X^2^ = 0.36, df = 2, p = 0.84) or “Policies and protocols reasonably match day-to-day work” (X^2^ = 1.08, df = 2, p = 0.58). Likewise, there was not a statistically significant difference in responses regarding self-doubt, fear of repercussion, or anxiety when bypassing a policy or protocol in order to meet the needs of a patient (X^2^ = 2.86, df = 2, p = 0.24), when strictly adhering to a policy or protocol despite the needs of a patient (X^2^ = 4.01, df = 2, p = 0.13), or when encountering a lack of policy or protocol (X^2^ = 1.09, df = 2, p = 0.58). The groups for 11-15 years, 16-20 years, and over 20 years were combined due to small individual group sizes (Table 6).

**Table 6:** Comparative analysis among years in clinical practice groups The Kruskal-Wallis test was used for analysis, and no statistically significant differences were detected for any of the survey prompts. Data is reported as the number of responses and percent of total responses (n (%)). Comparative statistics are reported as X2, df, and p-values. A p-value <0.05 was considered significant.

Comparative analysis among years in clinical practice groups						
“My work environment allows enough independence for me to adapt to the needs of patients or situations.”						
Response	0-5 years (%)	6-10 years (%)	11-15 years (%)	16-20 years (%)	>20 years (%)	Comparative analysis
Almost always	3 (23.08)	4 (22.22)	0 (0.00)	2 (50.00)	1 (33.33)	X^2^ = 0.36, df = 2, p = 0.84
Most of the time	10 (76.92)	12 (66.67)	3 (100.00)	2 (50.00)	1 (33.33)	
About half the time	0 (0.00)	1 (5.56)	0 (0.00)	0 (0.00)	1 (33.33)	
Sometimes	0 (0.00)	1 (5.56)	0 (0.00)	0 (0.00)	0 (0.00)	
Almost never	0 (0.00)	0 (0.00)	0 (0.00)	0 (0.00)	0 (0.00)	
Total	13 (100)	18 (100)	3 (100)	4 (100)	3 (100)	
Median (IQR)	4 (0)	4 (0)	4 (0.75)			
“Policies and protocols reasonably match day-to-day work.”						
Response	0-5 years (%)	6-10 years (%)	11-15 years (%)	16-20 years (%)	>20 years (%)	Comparative analysis
Almost always	3 (23.08)	3 (16.67)	0 (0.00)	2 (50.00)	1 (33.33)	X^2^ = 1.08, df = 2, p = 0.58
Most of the time	7 (53.85)	12 (66.67)	0 (0.00)	2 (50.00)	0 (0.00)	
About half the time	3 (23.08)	2 (11.11)	2 (66.67)	0 (0.00)	0 (0.00)	
Sometimes	0 (0.00)	1 (5.56)	1 (33.33)	0 (0.00)	2 (66.67)	
Almost never	0 (0.00)	0 (0.00)	0 (0.00)	0 (0.00)	0 (0.00)	
Total	13 (100)	18 (100)	3 (100)	4 (100)	3 (100)	
Median	4 (0)	4 (0)	3.5 (2.5)			
“I experience self-doubt, fear of repercussion, and/or anxiety when I bypass a current policy or protocol in order to meet the needs of a patient or situation.”						
Response	0-5 years (%)	6-10 years (%)	11-15 years (%)	16-20 years (%)	>20 years (%)	Comparative analysis
Almost always	3 (25.00)	0 (0.00)	1 (33.33)	0 (0.00)	2 (66.67)	X^2^ = 2.86, df = 2, p = 0.24
Most of the time	2 (16.67)	2 (11.11)	2 (66.67)	1 (25.00)	0 (0.00)	
About half the time	2 (16.67)	8 (44.44)	0 (0.00)	0 (0.00)	0 (0.00)	
Sometimes	5 (41.67)	5 (27.78)	0 (0.00)	2 (50.00)	0 (0.00)	
Almost never	0 (0.00)	3 (16.67)	0 (0.00)	1 (25.00)	1 (33.33)	
Total	12 (100)	18 (100)	3 (100)	4 (100)	3 (100)	
Median (IQR)	3 (2.25)	3 (1.00)	4 (2.75)			
“I experience self-doubt, fear of repercussion, and/or anxiety when I strictly adhere to a current policy or protocol despite the needs of a patient or situation.”						
Response	0-5 years (%)	6-10 years (%)	11-15 years (%)	16-20 years (%)	>20 years (%)	Comparative analysis
Almost always	2 (15.38)	1 (5.56)	1 (33.33)	0 (0.00)	0 (0.00)	X^2^ = 4.01, df = 2, p = 0.13
Most of the time	7 (53.85)	5 (27.78)	1 (33.33)	2 (50.00)	1 (33.33)	
About half the time	1 (7.69)	2 (11.11)	0 (0.00)	0 (0.00)	1 (33.33)	
Sometimes	3 (23.08)	8 (44.44)	1 (33.33)	1 (25.00)	0 (0.00)	
Almost never	0 (0.00)	2 (11.11)	0 (0.00)	1 (25.00)	1 (33.33)	
Total	13 (100)	18 (100)	3 (100)	4 (100)	3 (100)	
Median (IQR)	4 (1)	2 (2)	3.5 (2)			
“I experience self-doubt, fear of repercussion, and/or anxiety when I encounter situations not addressed by current policies or protocols.”						
Response	0-5 years (%)	6-10 years (%)	11-15 years (%)	16-20 years (%)	>20 years (%)	Comparative analysis
Almost always	1 (8.33)	0 (0.00)	0 (0.00)	0 (0.00)	0 (0.00)	X^2^ = 1.09, df = 2, p = 0.58
Most of the time	4 (33.33)	2 (11.11)	1 (33.33)	0 (0.00)	1 (33.33)	
About half the time	0 (0.00)	5 (27.78)	1 (33.33)	0 (0.00)	0 (0.00)	
Sometimes	5 (41.67)	8 (44.44)	1 (33.33)	2 (50.00)	1 (33.33)	
Almost never	2 (16.67)	3 (16.67)	0 (0.00)	2 (50.00)	1 (33.33)	
Total	12 (100)	18 (100)	3 (100)	4 (100)	3 (100)	
Median (IQR)	2 (2)	2 (1)	2 (1.5)			

When analyzing responses among genders, there were no statistically significant differences in responses to the prompts “My work environment allows enough independence for me to adapt to the needs of patients or situations” (W = 188, p = 0.83) or “Policies and protocols reasonably match day-to-day work” (W = 203, p = 0.83). Likewise, there was not a statistically significant difference in responses regarding self-doubt, fear of repercussion, or anxiety when bypassing a policy or protocol in order to meet the needs of a patient (W = 213.5, p = 0.46), when strictly adhering to a policy or protocol despite the needs of a patient (W = 195.5, p = 1.00), or when encountering a lack of policy or protocol (W = 167, p = 0.67) (Table 7).

**Table 7:** Comparative analysis among reported gender groups The Mann-Whitney U test was used for analysis, and no significant difference was detected for any of the survey prompts. Data is reported as the number of responses and percent of total responses (n (%)). Comparative statistics are reported as W-values and p-values. A p-value <0.05 was considered significant.

Comparative analysis among reported gender groups			
“My work environment allows enough independence for me to adapt to the needs of patients or situations.”			
Response	Female (%)	Male (%)	Comparative analysis
Almost always	6 (23.08)	4 (26.67)	W = 188, p = 0.83
Most of the time	18 (69.23)	10 (66.67)	
About half the time	2 (7.69)	0 (0.00)	
Sometimes	0 (0.00)	1 (6.67)	
Almost never	0 (0.00)	0 (0.00)	
Total	26 (100)	15 (100)	
Median (IQR)	4 (0)	4 (0.5)	
“Policies and protocols reasonably match day-to-day work.”			
Response	Female (%)	Male (%)	Comparative analysis
Almost always	7 (26.92)	2 (13.33)	W = 203, p = 0.83
Most of the time	11 (42.31)	10 (66.67)	
About half the time	6 (23.08)	1 (6.67)	
Sometimes	2 (7.69)	2 (13.33)	
Almost never	0 (0.00)	0 (0.00)	
Total	26 (100)	15 (100)	
Median (IQR)	4 (1.75)	4 (0)	
“I experience self-doubt, fear of repercussion, and/or anxiety when I bypass a current policy or protocol in order to meet the needs of a patient or situation.”			
Response	Female (%)	Male (%)	Comparative analysis
Almost always	5 (20.00)	1 (6.67)	W = 213.5, p = 0.46
Most of the time	3 (12.00)	4 (26.67)	
About half the time	8 (32.00)	2 (13.33)	
Sometimes	6 (24.00)	6 (40.00)	
Almost never	3 (12.00)	2 (13.33)	
Total	25 (100)	15 (100)	
Median (IQR)	3 (2)	2 (2)	
“I experience self-doubt, fear of repercussion, and/or anxiety when I strictly adhere to a current policy or protocol despite the needs of a patient or situation.”			
Response	Female (%)	Male (%)	Comparative analysis
Almost always	3 (11.54)	1 (6.67)	W = 195.5, p = 1.00
Most of the time	9 (34.62)	7 (46.67)	
About half the time	3 (11.54)	1 (6.67)	
Sometimes	9 (34.62)	4 (26.67)	
Almost never	2 (7.69)	2 (13.33)	
Total	26 (100)	15 (100)	
Median (IQR)	3 (2)	4 (2)	
“I experience self-doubt, fear of repercussion, and/or anxiety when I encounter situations not addressed by current policies or protocols.”			
Response	Female (%)	Male (%)	Comparative analysis
Almost always	1 (3.85)	0 (0.00)	W = 167, p = 0.67
Most of the time	5 (19.23)	3 (21.43)	
About half the time	2 (7.69)	4 (28.57)	
Sometimes	13 (50.00)	4 (28.57)	
Almost never	5 (19.23)	3 (21.43)	
Total	26 (100)	14 (100)	
Median (IQR)	2 (1)	2 (1)	

## Discussion

Increased understanding of “when things go right,” performance variability, and work-as-done are necessary for a Safety II approach to patient safety [2]. Due to the unpredictable, and therefore unprescribable, nature of patient care in the ED, this setting emerges as an ideal study environment to evaluate the current state of proactive safety through performance variability and graceful extensibility. To our knowledge, a similar study has not been done to date. As such, there is a lack of literature to which we can compare the results of this study.

Although human variability has been viewed as a liability in the healthcare setting, we report 127 instances where nuanced performance variability by healthcare team members contributed to patient safety. The largest category of reported instances included recognizing and intervening in an urgent patient need to promote safety and prevent harm. One response stated, “A patient was arriving with clinical presentation of an aortic dissection. The physicians were busy with a cardioversion next door. The writer and nursing team double-lined, drew labs, printed the EKG, and brought the ultrasound to the bedside. The physician was pulled from next door due to critical status. The FAST exam was positive. The patient went to the resuscitation bay.” A protocol for nursing orders for a chest pain patient only would have included obtaining an EKG and basic labs and assigning an Emergency Severity Index code. Nonetheless, this team worked outside of the protocol to establish two intravenous lines, bring the ultrasound to the bedside, and immediately alert the physician that they had a clinical concern about their patient. Our institution does not have a protocol in place to account for this unique, life-threatening scenario. However, the team applied their expertise and adapted to concurrent system stressors, thus promoting patient safety within the context of events unanticipated by the prescribed workflow.

Another way to view the submitted instances of proactive safety is to recognize them as instances wherein a hazard was prevented from progressing to harm. Hazards are factors that introduce a risk of harm within a sociotechnical system [9]. Efforts to improve patient safety may focus on identifying, eliminating, and mitigating the impact of hazards [10]. While the survey prompts in this study did not specifically reference hazard mitigation, respondents universally described scenarios in which there was a risk or hazard that was prevented from translating into harm because of clinician adaptations. By considering this perspective, we see that clinician adaptations mitigated the translation of hazards into harm, in contrast to the traditional perspective that would view practice adaptations as increasing the risk of harm.

Survey respondents also described situations when they bypassed a policy or protocol to meet the needs of a patient or situation. Most respondents described bypassing a policy or protocol to meet the physical or social needs of their patient. For example, one respondent described the following event: “A patient wouldn’t stay for stroke workup unless they could smoke. I let them go outside and smoke.” The patient and the care team had the same ultimate goal, to obtain the necessary stroke evaluation and management. However, the patient’s nicotine dependence, in combination with the hospital policy of patients not leaving the ED prior to admission once care is started, posed a barrier. While policy would state that they discharge the patient against medical advice, the care team recognized that the patient needed continued medical care. In bypassing the policy, the care team prioritized the patient’s need for stroke care. Policies and protocols function to keep patients safe. However, given the complexity of our clinical care environments and our aim to take optimal care of unique and individual human patients, care cannot be prescribed to the exclusion of nuanced decisions made at the bedside. In an ideal situation, this patient would not smoke, a risk factor that likely led to their stroke. However, unlike the industries from which Safety I was adopted, healthcare involves imperfect human beings and nuanced situations, thus requiring real-time adjustments to achieve desired outcomes.

Most respondents reported that policies in the ED reasonably match their day-to-day work and that they felt supported in adapting to the needs of patients and situations. While we did not detect the difference we expected between work-as-imagined and work-as-done, comments from respondents support its existence. Two qualitative responses specifically preface a described instance with statements such as “I am having a hard time answering this question. I feel that this is something we do every day.” and “This is the job description of a nurse. I do this every day.” Hollnagel discusses the difficulty in measuring everyday work that results in desired outcomes. He proposes that because these events are frequent, small-scale, and result in a desired outcome, they are often ignored and difficult to measure [2]. We suspect that this lack of perceived difference between work-as-imagined and work-as-done on Likert Scale data results from respondents being acclimated to frequent small deviations from work-as-imaged. As stated well by one of the respondents, “There are many times I break ‘the rule;’ not every case or patient is the same.” Care team members in our ED recognize core concepts of Safety II and graceful extensibility playing out in their everyday work, even if they do not know the theoretical background. There is a sense of the necessity and ubiquity of performance variability to adequately care for patients in the ED.

We know that well-intended policies and procedures are sometimes handed down without input from frontline personnel or consideration for the nuances of providing safe emergency care. Yet, these results demonstrate that team members think that policies reasonably match daily work and feel supported to employ real-time adaptations. Any gap between work-as-imagined and work-as-done is likely mitigated by ED care team involvement in protocol implementation. Results may reflect a larger gap between work-as-imagined and work-as-done if we had asked about a specific policy rather than considering “policies and procedures” en masse. Further, most of the examples reported are not adaptations that would be frowned upon by management but rather lauded. Although ED care team members are subject to many protocols, adaptability is still a widespread expectation of work in the ED. Therefore, expectations of strict adherence to internal policies or procedures as proposed by Safety I may not exist in the ED to the same extent as in other clinical practice environments.

Respondents report experiencing self-doubt, fear of repercussion, or anxiety most frequently when strictly adhering to a policy despite the needs of a patient or situation. This may stem from the inherent sense that adapting to patient needs is an expected part of their daily work. Prior research shows that common workplace motivators in emergency care team members include teamwork, pride, a unique skill set, and helping patients in a time of need [11]. This may contribute to fewer respondents reporting frequent negative emotions when utilizing their expertise and skill to help meet the needs of their patients than when strictly adhering to a policy despite the needs of their patients.

Hollnagel advocates for a mixed approach, where components of Safety I and Safety II are necessary to optimize patient safety and minimize patient harm [2]. While policies and protocols exist to keep patients safe, no protocol can address every iteration of a patient presentation, especially in an unpredictable clinical environment such as the ED. There are no doubt roles for protocols in certain situations, and much is context dependent. For example, the use of order sets for diabetic ketoacidosis, sepsis, or neutropenic fever prevents the risk of lapses when ordering in an environment characterized by frequent interruptions. However, they are just tools. Their role in safety is finite and, although necessary, is not sufficient. To advance patient safety, we must have a balanced approach both in clinical work and in interpreting the data for this study. We do not suggest that patients would be safer if policies and protocols were never followed. Rather, we interpret these findings as reflective of a positive culture of safety and demonstrating key concepts of Safety II.

Limitations

While the data collected in this study clearly demonstrate the presence of a culture of Safety II in our ED, there are some inherent limitations to this study. This is a voluntary, survey-based study. Therefore, it may be limited by sampling or non-response bias. We invited all ED care team members to participate in the study, therefore limiting sampling bias. Nonetheless, care team members who could not recall an instance of performance variability may have decided not to respond to the survey at all. In analyzing qualitative data, we did not endeavor to report on the rate or incidence of proactive safety but rather to describe instances where it occurred. Likert data may be more likely to reflect positive impressions of support and policies matching day-to-day work, as respondents who are dissatisfied with their work may be less likely to engage in a voluntary workplace survey. Further, we did not specify a time frame during which to consider how well policies and procedures matched daily work. Given the dynamic nature of the clinical practice environment and the potential for changes to hospital policies or procedures, our cross-sectional evaluation of this represents only current state perspectives at the time of the study. These limitations should be considered when interpreting this data. To our knowledge, no prior studies have posed these questions to ED care teams. We cannot compare our findings to prior research.

When examining practice variation, we have historically done so in the setting of reviewing a case with a known poor outcome. This creates a false perception of a linear correlation between practice variation and bad outcomes. The approach does not highlight, collect, or review when clinicians act outside of protocol and desired outcomes are achieved. This creates a literature gap, requiring further exploration of the resilient actions of staff. Nonetheless, this study may have the opposite effect: staff recall instances when they exercised practice variability, and the outcome was positive. The reality likely falls somewhere in between. In fact, the same systems and patterns of practice that result in things going wrong often result in things going right [2]. A true understanding of what causes the difference in outcome requires further multidisciplinary investigation.

This study was conducted at an academic level 1 trauma center ED, where teams include learners at varying levels of medical training. Therefore, results may lack generalizability to non-academic centers. Further, the tertiary care setting may introduce higher clinical complexity, acuity, and patient volumes than an ED in the community setting. However, teamwork in healthcare always exists. Even in a remote, critical access ED, there will be a team of physicians, a nurse, and likely at least one additional technician. Anywhere that teams function to provide patient care, concepts of Safety II apply. Further research is necessary in various ED sizes and settings.

Further, commonly reported instances of time-based performance adjustments, team-based collaboration, and support for performance variability may reflect the clinical practice environment of the ED. Patients arrive undifferentiated, and care teams work in close physical and temporal proximity to urgently diagnose and treat potentially life-threatening conditions. Research outside of an ED clinical practice environment is also necessary, as performance variability themes, support for adaptability, and other experiences of Safety II may differ in an alternative setting such as an inpatient unit or outpatient clinic.

This study asked respondents to think about and provide information about times they deviated from policy or procedure or were involved in or witnessed potential patient harm. This may have dissuaded some team members from responding or provoked fear or repercussion, or self-doubt. To minimize these limitations, the survey was anonymous and was sent to all care team members by a human factors engineer in the hospital quality office, an impartial third party without influence on performance evaluations, promotions, or remediation decisions.

Inherent in a survey-based study on a complex concept such as Safety II is the tendency to oversimplify reality. Safety II itself asserts that the nuances and complexities of the clinical practice environment are dynamic, ever-changing, and often indescribable in retrospect. Therefore, any retrospective, cross-sectional evaluation of the Safety II phenomenon may similarly fall short of representing the true complexities of any scenario and the current state of Safety II as a whole. For example, the specific reasons for bypassing policy and the outcomes of adaptive actions were not examined in this study. Nonetheless, this study represents a necessary first step in understanding Safety II in our current clinical practice environments. Further research, such as utilizing interviews or focus groups, is necessary to examine the intricacies of the Safety II experience of care team members.

## Conclusions

In our tertiary care, academic ED, team members kept patients safe through collaboration, time-sensitive interventions, physical harm prevention, and interactions with patients and visitors. They bypassed policies when necessary to meet the physical and social needs of patients. Most team members felt that existing policies matched day-to-day work and that they were supported in adapting to the needs of patients. Respondents reported experiences of self-doubt, fear of repercussion, or anxiety most often when adhering to a policy despite the needs of a patient or situation. This work stands as a sampling of resilient, expertise-driven actions of frontline staff in the ED and serves as a call for further exploration into the practical impact of safety II principles and work as done in the emergency setting.

## Appendices

Appendix A

**Table 8:** Anonymous survey distributed to members of the emergency department care team This table presents the survey response prompts along with the available response options. No data is reported in this table.

Survey prompt	Response options
What is your role on the healthcare team?	Technical/technologist
	Nurse
	Advanced practice provider
	Attending physician
	Resident or fellow physician
	Therapist (RT/PT/OT/ST)
	Pharmacist
	Social worker/case manager
	Medical student
	Other clinical role
	Other non-clinical but patient-facing role
What is your age (years)?	<20
	20-29
	30-39
	40-49
	50-59
	60-69
	>70
How many years have you been working in any clinical setting?	0-5
	6-10
	11-15
	16-20
	Over 20
What is your gender?	Female
	Male
	Non-binary
	Prefer not to describe
	Other
Please tell us about a time when you or a team member prevented any amount of harm from occurring to a patient.	Free text response
Please tell us about a time when you or a team member had to bypass current policies or protocols to keep a patient safe.	Free text response
Please respond to the prompts below regarding your current practice environment.	
My work environment allows enough independence for me to adapt to the needs of patients or situations.	Almost always
	Most of the time
	About half the time
	Sometimes
	Almost never
Policies and protocols reasonably match my day-to-day work.	Almost always
	Most of the time
	About half the time
	Sometimes
	Almost never
	Almost always
Please respond to the prompts below regarding your personal experience. I experience self-doubt, fear of repercussion, and/or anxiety when:	
I bypass a current policy or procedure in order to meet the needs of a patient or situation.	Almost always
	Most of the time
	About half the time
	Sometimes
	Almost never
I strictly adhere to a current policy or protocol despite the needs of a patient or situation.	Almost always
	Most of the time
	About half the time
	Sometimes
	Almost never
I encounter situations not addressed by current policies or protocols.	Almost always
	Most of the time
	About half the time
	Sometimes
	Almost never
